# MKRN3 role in regulating pubertal onset: the state of art of functional studies

**DOI:** 10.3389/fendo.2022.991322

**Published:** 2022-09-16

**Authors:** Stefania Palumbo, Grazia Cirillo, Francesca Aiello, Alfonso Papparella, Emanuele Miraglia del Giudice, Anna Grandone

**Affiliations:** Department of Child, Women, General and Specialized Surgery, University of Campania, Naples, Italy

**Keywords:** MKRN3, central precocious puberty, functional studies, E3 ubiquitin ligase, epigenetic, auto-ubiquitination

## Abstract

Puberty is a critical process characterized by several physical and psychological changes that culminate in the achievement of sexual maturation and fertility. The onset of puberty depends on several incompletely understood mechanisms that certainly involve gonadotropin-releasing hormone (GnRH) and its effects on the pituitary gland. The role of makorin ring finger protein 3 (MKRN3) in the regulation of pubertal timing was revealed when loss-of-function mutations were identified in patients with central precocious puberty (CPP), which to date, represent the most commonly known genetic cause of this condition. The *MKRN3* gene showed ubiquitous expression in tissues from a broad spectrum of species, suggesting an important cellular role. Its involvement in the initiation of puberty and endocrine functions has just begun to be studied. This review discusses some of the recent approaches developed to predict MKRN3 functions and its involvement in pubertal development.

## Introduction

Puberty is a complex developmental process through which organisms acquire sexual maturity, characterized by the acquisition of secondary sexual characteristics, the maturation of the gonads, and the attainment of reproductive capacity (1). From a purely neuroendocrine perspective, puberty begins with an increased pulsatile release of GnRH from neurosecretory neurons, scattered from the pre-optic area (POA) to the caudal hypothalamus, which induces increased production of LH and FSH by the pituitary gland necessary for gonadal function. In humans, GnRH has a pulsatile secretion during the fetal life and mini-puberty, followed by a period of quiescence during childhood and a reactivation of its secretion at puberty (2). Systems biology approaches have recently suggested that the control of puberty and its timing is regulated by multiple sets of genes/proteins and requires the involvement of several mechanisms that can coordinate the hierarchical activation/deactivation of stimulatory and inhibitory neuronal pathways (3). A considerable number of studies over the past decades have investigated these neural networks, and several neuropeptides and neurotransmitters have been identified in the intricate balance between inhibitory and excitatory inputs to GnRH neurons. Among these, kisspeptin (encoded by the *KISS1* gene), neurokinin B (encoded by the *TAC3* gene) and glutamate exert excitatory functions and are critical for pubertal activation of GnRH neurons. Loss of function mutations in some of these neurotransmitters or alteration in their signaling lead to hypogonadotropic hypogonadism, while very rare gain of function mutations cause early reactivation of GnRH secretion and central precocious puberty (CPP) (4).

CPP is characterized by the gonadotropin-dependent development of secondary sexual characteristics before the age of 8 in girls and 9 years in boys. In 2013, loss of function mutations in *MKRN3*, a maternally imprinted gene that encodes makorin RING-finger protein-3, have been identified in five families with CPP unrevealing a new inhibitory component of the gene regulatory network that governs the onset of puberty (5). In particular an increasing number of deleterious mutations has been described in both familial and apparently sporadic cases, making MKRN3 deficiency the most frequent genetic cause of central precocious puberty with a prevalence ranging from 0.5-17.5% in sporadic cases to 9-46% in familial cases (6, 7). The human *MKRN3* gene is composed of a single exon and is located on the 15q11.2-q13 chromosome, in the critical region of Prader Willi syndrome and it is subjected to maternal imprinting. Recent genome-wide association studies (GWAS) reported associations between several paternally inherited *MKRN3* variants and the age at menarche, highlighting its important role during puberty onset (8). Furthermore, a peripubertal decline in the serum levels of MKRN3 has been documented in both sexes (9–11) and different studies have shown that the hypothalamic expression of MKRN3 mRNA and protein is significantly reduced before the onset of puberty in mice, suggesting MKRN3 potential relevance of the repressive actions in the central control of puberty (5). Although the above evidence collectively supports a relevant inhibitory role of MKRN3 in pubertal onset, the molecular mechanisms and/or regulatory elements responsible for its precise control remain unclear. This review presents a state-of-the-art of the *in vivo* and *in vitro* functional studies focusing on MKRN3 potential mechanisms of action.

## MKRN3 protein structure and expression in animals and humans

*MKRN3* was the first member of the makorin gene family identified in 1999 by Jong et al. (12) as one of several maternally imprinted genes located in the critical region of Prader-Willi syndrome (PWS) of human chromosome 15q11.2-q13.

Like all other makorin proteins, MKRN3 has a distinctive organization. Specifically, MKRN3 consists of a centrally located RING finger motif (C3HC4), two amino-terminal C3H zinc finger motifs followed by a unique pattern of conserved Cys-His residues called Makorin zinc finger motif, and a carboxy-terminal C3H zinc finger motif. From this specific structure, MKRN3 functions can be predicted. The function of the unique Cys-His makorin motif is still unknown, but it has been suggested to be a DNA binding domain. The C3H zinc fingers have been found in a variety of ribonucleoproteins suggesting an RNA-binding function that can be involved in post-transcriptional RNA processing at multiple levels, including alternative splicing, mRNA stability, mRNA localization and translation efficiency. The RING finger domain is found in most E3 ubiquitin ligases and mediates protein enzymatic activity of the protein by transferring ubiquitin from an E2 ubiquitin-conjugating enzyme to target protein substrates (13). These modifications have a range of biological effects on the target protein substrate, from proteasome-dependent proteolysis to post-translational control of protein function, structure, assembly, and/or localization. In fact, E2-E3 complexes can monoubiquitinate a lysine substrate altering the cellular non-proteolytic functions by changing protein stability or localization or synthesizing a polyubiquitin chain of lysine residues driving proteolytic processes (14, 15).

**Figure 1:**
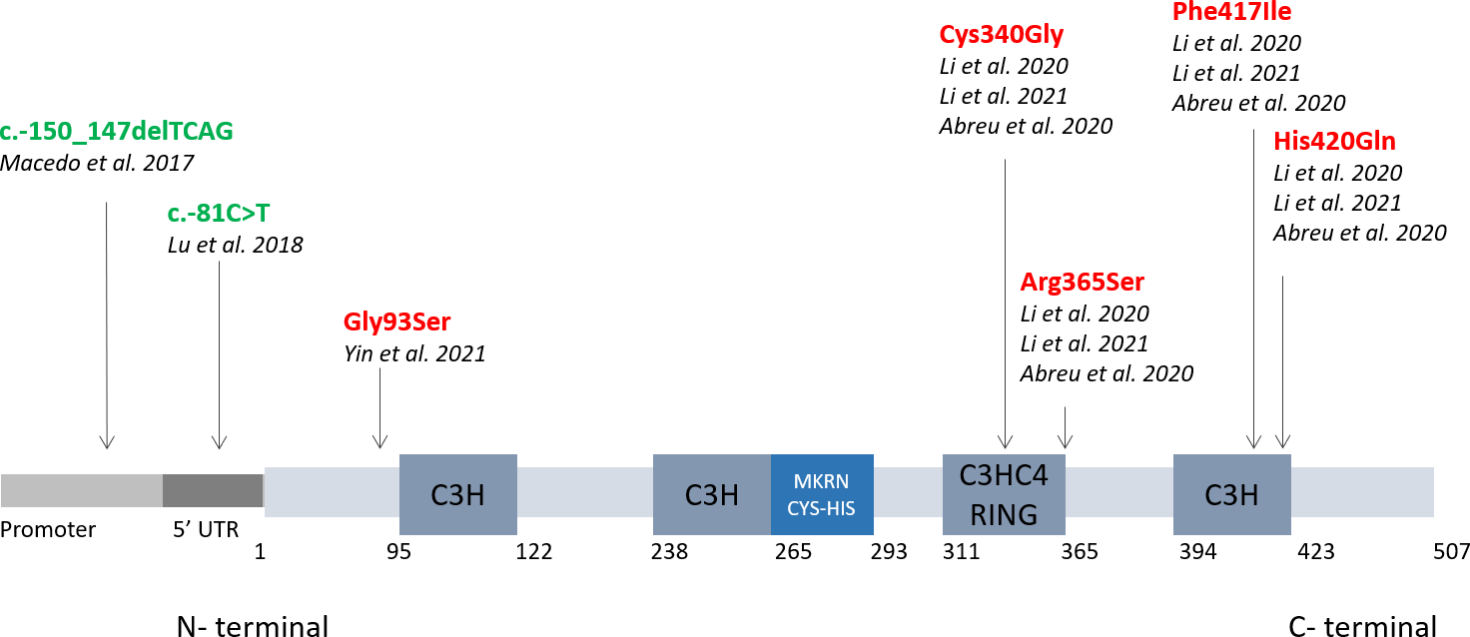
CPP-associated mutations compromise the auto-ubiquitination and binding affinity of MKRN3. The image shows the structure of the MKRN3 gene, which consists of three zinc finger domains (C3H) and one RING finger domain (C3HC4). The major mutations tested in functional studies are shown here with a black arrow indicating their location on the MKRN3 protein. Mutations causing defects in MKRN3 expression are shown in green, while in red are shown mutations affecting auto-ubiquitination or binding of MKRN3 to promoters of effector genes.

MKRN3 is highly conserved between species, in fact amino acid sequences in mice and humans share approximately 69% identity and 82% similarity with the highest level in the RING and 3’ UTR region implying important functional significance for these regions of the protein (12, 16). Although MKRN3 is ubiquitously expressed in adult human tissues, a previous study in mice showed that Mkrn3 is highly expressed in the hypothalamic arcuate nucleus (ARC), where a group of neurons, named KNDy (Kisspeptin/Neurokinin/Dynorphin), are located and considered critical regulators of GnRH secretion (**Figure 1**). The inverse correlation between *Mkrn3* expression and *Kiss1* and *Tac3* in ARC before the onset of puberty suggests that Mkrn3 may play a role in the inhibition of GnRH secretion during the quiescent prepubertal period, probably acting at the hypothalamic level to inhibit stimulatory inputs (17). Phenotypic studies have shown that *Mkrn3* knockout mice present many symptomatic features of human CPP while also showing 50% higher expression of GnRH1 mRNA than wild-type controls. In GT1-7 cells, derived from GnRH-positive hypothalamic neurons, mouse MKRN3 potently repressed GnRH1 expression at both mRNA and protein levels (18). This inhibition may be due to ubiquitin-dependent protein degradation mechanisms or the direct involvement of Mkrn3 in transcriptional repression, as already demonstrated for Mkrn1, which appears to inhibit RNA polymerase II-dependent activators through a transcriptional interference or “squelching” process (19).

Furthermore, data reported by Human Protein Atlas indicate that MKRN3 is expressed mainly in the plasma membrane and in the cytoplasm, but also in the nucleus. Based on its location in the plasma membrane, it can be speculated that MKRN3 may also be involved in endocytosis and receptor down regulation, as has been shown for some other E3 ligases (20).

## Function through mutations effect

### Phenotype of patients with MKRN3 mutations

The MKRN3 mutations are the most common form of familial CPP. A recent study by Seraphim et al. summarized the phenotype of girls and boys with MKRN3 defects (21).

The clinical picture is indistinguishable from idiopathic CPP. The median age at the time of onset in girls is 6.2 ± 1.2 years while in boys it is 7.1 ± 1.5 years, confirming data from a recent meta-analysis and the study by Bessa focusing on male CPP (6, 22). Patients with CPP caused by MKRN3 defect had higher levels of FSH compared to those with idiopathic CPP.

Regarding the genotype/phenotype correlation, severe mutations (stop codon or frame shift mutations) were associated with greater bone age advancement than missense variants (21).

Finally, studies that reported data on adult patients with MKRN3 variants treated with GnRH analogs showed a good response to treatment (21, 23).

### Ubiquitination and autoubiquitination activity

Ubiquitin modification of many cellular proteins targets them for proteasomal degradation. A notable feature of RING E3 ubiquitin ligases is that the enzymatic activity of the E3 ligase can be monitored through ubiquitination of the protein *in vitro*. In fact, as well as promoting the addition of ubiquitin residues on target proteins, E3 ligases regulate their own stability within the cell through auto-ubiquitination, the process by which E3 enzymes catalyze the addition of polyubiquitin to themselves. In this context, MKRN3 could be considered a specific target because it contains a RING finger motif that has an active site for an E3 ubiquitin ligase involved in both autoubiquitination and substrate ubiquitination reactions (15, 24).

**Figure 2:**
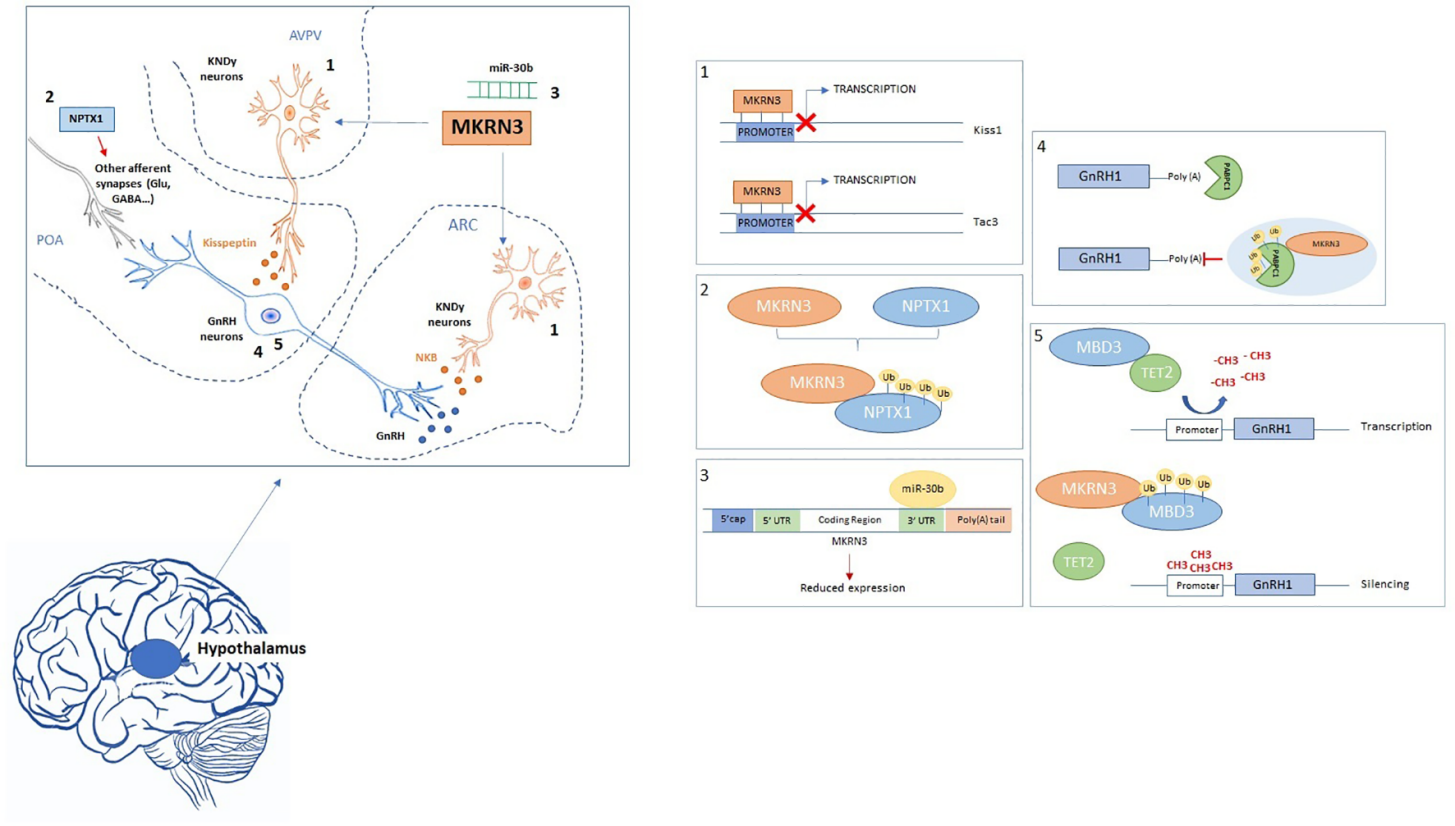
Schematic picture of MKRN3 interactions. The figure summarizes the neuronal mechanisms of action of MKRN3 and its effectors. The insert on the left shows the hypothalamus and the different nuclei that are encircled by dotted blue lines. The GnRH neuron is depicted in blue while KNDy (Kisspeptin-Neurokinin-Dynorphin) neurons are shown in orange. In the inserts on the right are represented the mechanisms of action of MKRN3 on its targets. The number of each insert report the position of the interaction on the hypothalamic neurons. Red X indicates inhibitory action. POA, preoptic area; AVPV, anteroventral periventricular nucleus; ARC, arcuate nucleus.

To this end, Li et al. (18) assembled an *in vitro* ubiquitination system to contain ATP, an E1 Ub-activating enzyme, MKRN3 as E3 ligase and different E2 Ub conjugating enzymes to support MKRN3 auto-ubiquitination. Affinity purification assays indicated that the recombinant MKRN3 protein interacted directly with E2 enzymes supporting MKRN3 autoubiquitination. Diagnostic bands detected for MKRN3 by immunoblots or staining with Coomassie Blue on gel showed a profile of MKRN3 compatible with ubiquitinated proteins, similar to a smear. Thus, it was clear that wild-type *MKRN3*, like many other RING-type E3 ligases, could undergo autoubiquitination. When similar *in vitro* ubiquitination reactions were performed with wild-type MKRN3 or mutants (Cys340Arg, Arg365Ser, Phe417Ile, His420Gln) (**Figure 2**), it was evident that disease-causing mutations in the RING domain or C-terminal region compromised autoubiquitination of MKRN3 (18). These data suggested that CPP-associated MKRN3 mutations may stabilize the MKRN3 protein by altering the activity of E3 ligase (17, 18, 25). This would lead to a subsequent reduction in autoubiquitination and a slower proteasome-mediated degradation. In addition, MKRN3 mutants showed weaker suppression of GnRH1 promoter transcriptional activity than MKRN3 wild-type (18). The same MKRN3 mutations associated with CPP were tested by Abreu et al. who showed similar expression levels both for wild-type or mutants into HEK293T cells after plasmids transfection of wild-type and mutants MKRN3 and subsequent western blot analysis. Interestingly, a dramatic decrease in ubiquitination was observed in MKRN3 mutations localized in the RING finger (Cys340Arg, Arg365Ser) compared to a weaker decrease for mutants localized in the zinc finger (Phe417Ile, His420Gln), even with similar levels of immunoprecipitated protein, highlighting the importance of the integrity of the RING finger in E3 ubiquitin ligase activity (17).

Thus, autoubiquitination can lead to proteasome-dependent degradation and can be considered an essential regulatory mechanism that adds another piece to the understanding of MKRN3 homeostasis

### Defects causing reduced *MKRN3* expression

Most published studies describe causative mutations in the coding region of the *MKRN3* gene, and only a few studies recently reported defects in the regulatory region. In 2018, two groups identified in patients with CPP a small deletion (c.-150_-147delTCAG) in the promoter region of the *MKRN3* gene (26) and a single nucleotide substitution in the 5’ UTR(c.-81C>T) (27) both associated with a reduced promoter activity of the gene in transfected cells (**Figure 1**). Accordingly to these data, regulatory region of *MKRN3* was started to be screened and new mutations have been identified confirming the causative of puberty onset. *In silico* analysis has predicted that these mutations may lead to the formation of new binding sites for transcription factors (e.g. SOX4) or their loss (PRDM14; HMX2; MTE; DREAM) with a consecutive reduction in the expression levels of MKRN3 associated phenotypically with CPP (26, 28).

### MKRN3 and its targets

The mechanism of action of MKRN3 in the onset of puberty is still unclear, but some studies have begun to report investigations into possible targets of its action.

Mass spectrometry analyses performed on a stable HEK cell line expressing MKRN3 revealed 81 novel protein interaction partners of MKRN3 that are involved in various cellular processes such as insulin signaling, RNA metabolism, and cell-cell adhesion. Among these, 20 interactors, including LIN28B, have previously been associated with age at menarche in genome-wide association studies (8). Although LIN28B appears to influence the age of menarche and infant growth, the mechanism of interaction with MKRN3 during the timing of puberty is unknown. In rats and nonhuman primates, Lin28b expression decreases in the hypothalamus at puberty, while in mice the expression of both Lin28b and Mkrn3 is reduced before puberty, speculating that it may act in concert with Mkrn3 during this stage of development. MKRN3 has also been found to interact with OTUDS, a deubiquitinase protein linked to congenital hypogonadotropic hypogonadism that can act by counteracting the effects of MKRN3 during puberty. Unfortunately, to date, there are still few functional studies on the mechanism of action of MKRN3 on its effectors.

In the following, we report the better investigated interactions.

### Direct action of Mkrn3 on expression of Kiss1, Tac3 and Gnrh1

The onset of pubertal development is a genetically controlled mechanism due to activation of the HPG axis and a subsequent increase in GnRH secretion through a complex neuronal network.

Surprisingly, while the players involved in the stimulation of GnRH neurons in puberty have been studied extensively, it is only very recently that inhibitors of GnRH, including MKRN3, have been identified.

Due to the relative inaccessibility of the human brain, the functional neuronal studies have been conducted in animal models or in engineered neurons. Using the CRISPR/Cas9 approach, Yellapragada et al. generated human-induced pluripotent stem cell (hiPSC) knockout lines for *Mkrn3* that subsequently have been differentiated into GNRH-expressing neurons. Analysis of GnRH1 expression levels showed no difference between wild-type and MKRN3-deficient cells, leading to the hypothesis that Mkrn3 is dispensable for GnRH neuron differentiation and GnRH1 expression (29). Other studies reported Mkrn3 expression may be localized into other neuronal districts involved in pubertal development and that its inhibitory action is played indirectly on GnRH secretion. Specifically, the arcuate nucleus (ARC) plays a key role in puberty control and exerts this effect through neurons that produce two key GnRH secretagogues: kisspeptin (encoded by *KISS1/Kiss1*) and neurokinin B (NKB, encoded by *TAC3/Tac3*) (30). In rodents and nonhuman primates, *in situ* hybridization showed coexpression of Kiss1 and Tac3 in ARC neurons and an increase in their mRNA levels during pubertal development has been reported (17). In parallel, the significant decrease in Mkrn3 levels in the same neurons suggests that Kiss and Tac3 may be two possible effectors of Mkrn3 inhibitory activity (17). A series of luciferase assays were performed in Neuro-2a cells transfected with vectors in which luciferase expression is driven by the human *KISS1* or *TAC3* promoter, with or without an expression vector encoding human MKRN3. Transfection of either promoter resulted in increased luciferase activity compared to the empty vector, and this activity was significantly reduced (40%-60%) by coexpression of MKRN3. MKRN3 inhibition of the activity of the *KISS1* and *TAC3* promoters did not affect the activity of the promoters of other genes involved in the stimulatory (*EAP1, Ttf1* and *Vglut2*) or inhibitory (*Viaat, Eed* and *PDYN*) control of puberty. MKRN3 then selectively represses the promoter activity of *KISS1* and *TAC3*, acting on Kiss1 neurons to reduce kisspeptin and NKB release and decrease GnRH secretion (**Figure 1**). Additional assays and chromatin immunoprecipitation tested the effects of some CPP-associated MKRN3 missense mutations (Cys340Arg, Arg365Ser, Phe417Ile, His420Gln) (**Figure 2**) revealing that only mutations located in the RING finger domain (Cys340Arg, Arg365Ser) impaired the ability of MKRN3 to inhibit the activity of *KISS1* and *TAC3* promoters but not its recruitment to these promoters.

One of the mutations located in the C-terminal domain of MKRN3 (Phe417Il) (**Figure 1**) showed instead a reduced binding affinity to the promoters of *KISS1* or *TAC3*, although without compromising or partially compromising its inhibitory activity. Structural analysis by X-ray crystallography and *in silico* prediction showed the intensity of the effects of different mutations in the C-terminal region of the gene, showing how a single amino acid change can alter the zinc finger conformation and its binding ability (17).

A recent mutation (Gly93Ser) reported in the N-terminal region (Fig1) showed reduced transcriptional activity of human *GNRH1, KISS1* and *TAC3* (25) suggesting an important role for the N-terminal in binding regulatory regions of genes as well. In addition, attenuated autoubiquitination activity has been reported, highlighting that this terminal region may also be involved in the ubiquitination process.

### MKRN3 and NPTX1

The Neural prentraxin-1 precursor (Nptx1) is an important protein for neuronal development and is highly expressed in the hypothalamus at the onset of puberty (31, 32) whereas a Mkrn3 decrease is observed (33). Moreover, Cummings et al. (34) reported that NPTX1 is expressed at glutamatergic synapses where it modifies glutamate release in the CNS. In that study, glutamate release increased with acute application of NPTX1. The excitatory amino acid glutamate and its N-methyl-D aspartic acid (NMDA) subtype receptor are important in the neural system that regulates sexual maturation (35). High- and multiple-dose injections of NMDA induce early puberty in rats, monkeys, and sheep. Modulation of Nptx1 levels could probably trigger this reaction thus leading to a precocious onset of the puberty. Although to date there is no direct evidence of the Nptx1 involvement in pubertal processes, the results reported by Liu et al. (33) showed a dynamic change in Nptx1 expression at different stages of the hypothalamus, indicating that Nptx1 may respond to the induction of GnRH neurons when puberty begins. Mass spectrometry and coimmunoprecipitation studies reported an interaction of Mkrn3-Nptx1 in the hypothalamus of 4-week-old mice after cerebroventricular injection of Flag-labeled Mkrn3. The RING finger domain appears to be essential for this interaction that is indeed not observed in a co-immunoprecipitation experiment using a mutant construct of Mkrn3 lacking the RING domain. The study also showed a reduction in the level of Nptx1 polyubiquitination in the hypothalamus of mice injected intracerebroventricularly with the mutant vector Mkrn3 deficient in the RING domain compared to control animals injected with the vector encoding wild-type Mkrn3. These data suggest that Mkrn3 may be able to modulate the level of Nptx1 through its E3 ubiquitin ligase activity (**Figure 1**) (33). The decrease in MKRN3 could therefore regulate NPTX1 activity in the hypothalamus resulting in increased glutamate release that could lead to early puberty. The interaction of these molecules appears to be a plausible hypothesis for the onset of puberty at the central level, although a recent study did not report a correlation of the level of MKRN3 and NPTX1 in peripheral blood in girls with CPP (36).

### MKRN3 and post-transcriptional modification: interaction with PABP

Recently, the critical role of MKRN3 in the regulation of post-transcriptional mechanisms involved in the initiation of puberty in mammals has emerged.

The group of Li et al. (37), identified poly(A)-binding proteins (PABPs) as potential targets of MKRN3 ubiquitination activity. PABPs are family of proteins consisting of a poly(A)-binding C- terminal domain and four RRM (RNA recognition motif) domains with different binding affinity to the poly(A) tail present at the 3’ end of mRNAs. This interaction appears to regulate many aspects of mRNA homeostasis, such as stability or non-nonsense-mediated decay (NMD), stress response, control of translation initiation, and mRNA translocation (38–40).

By HEK-293 cells transfected with Flag-tagged MKRN3 and subsequent co-IP assays and mass spectrometry analysis, PABP family members, particularly PABPC1, PABPC3 and PABPC4 were shown to form complexes with MKRN3. Subsequently, GST pull-down assays and mapping-analysis showed how the central region (126-295aa) of MKRN3 interacts directly with the C-terminal region of PABPC1 (37).

Confirmation that PABPs are real substrates of MKRN3 and not mere binding partners comes from the ability of MKRN3 to conjugate poly-ubiquitin chains on PABPC1, PABPC3, and PABPC4, whereas CPP-associated mutations (Cys340Arg, Arg365Ser,His420Gln) that fall in the RING of MKRN3 or C-terminal neighboring regions have impaired this ability, with the exception of the mutant (P417). Further *in vivo* ubiquitination assays then identified four Lys residues (312, 512, 620, and 625) in PABPC1 as the major sites for MKRN3-mediated ubiquitination. Additionally, the poly-Ub chains that MKRN3 conjugates to PABPC1, could disrupt the binding of PABPC1- poly(A) through the creation of a steric hindrance, rather than directly ubiquitinating the poly(A) binding motifs as assumed for the K27 and K29 residues. All ubiquitination sites were highly conserved in different organisms (human, mouse and rat), suggesting that MKRN3-mediated ubiquitination could also play a role in the regulation of other PABPs of different species as well (37) (**Figure 1**).

Dual luciferase reporter assays then showed higher GNRH1 levels in MKRN3 -/- HEK293 or MKRN3 mutant constructs than in wild-type. In addition, the presence of PABPC1 appears to regulate GNRH1 luciferase expression (UTR) by stabilizing its mRNA, an effect that was almost completely abolished by reintroduction of wild-type MKRN3 but not by the CPP-associated mutant (C340G) (37).

RNA immunoprecipitation (RNAIP) and Poly (A) tail length assays collectively clearly showed that ubiquitination of PABPC1 or PABPC4 by MKRN3 negatively regulates the formation of translation initiation complex (TIC), attenuating their binding to poly (A) tail-containing mRNAs and leading to shortening of the poly(A) tail of GNRH1 mRNA (37). This could contribute to higher levels of GNRH1 mRNA and protein when *MKRN3* was mutated in CPP patients, ablated, or silenced in mice at the beginning of puberty.

## MKRN3 and the epigenetic regulation of pubertal timing

In addition to genetic determinants, epigenetic mechanisms have recently emerged as important regulators of puberty onset, as suggested from experimental models. The elucidation of such mechanisms is still in its infancy, and, consequently, its pathophysiological implications in terms of perturbations of puberty (especially in humans) have yet to be fully characterized. Since the first half of the 1970s, DNA methylation has been described as a key epigenetic modification event involved in gene silencing. Briefly, DNA methylation and demethylation are catalyzed by DNA methyltransferases (DNMTs) and demethylases (human ten-eleven translocation methylcytosine dioxygenases, TETs), respectively. Such reactions occur at the carbon-5 position of cytosine residues in CpG nucleotides, leading to the formation or decomposition of 5-methylcytosine (5mC), 5-hydroxylmethylcytosine (5hmC) and other derivatives, which constitutes a fundamental epigenetic mechanism that regulates gene expression in mammalian cells (41). The DNA methylation profile results in a highly dynamic regulation of post-transcriptional modifications; increased methylation of 5-mC is associated with the gene promoter repression, while hypomethylation is associated with transcriptional activation. In mammalian genomes, most DNA methylation (about 70%) occurs on CpG island (42, 43). This epigenetic regulatory system is expressed in hypothalamus neurons involved in stimulating GnRH release, acting according to a transcriptional repression mechanism. In fact, in 2013, Lomniczi et al. identified a group of transcriptional silencing proteins, the Polycomb group (PcG), that repress genes responsible for the onset of puberty by preventing its premature onset (44). Increased methylation of PcG gene promoters and their reduced hypothalamic expression impair this repressive action, leading to activation of the *Kiss1* gene, thus demonstrating that in female rats the onset of puberty is regulated by epigenetic repression of the PcG complex (44).

Subsequently, experiments conducted in the basal hypothalamus using cultured GnRH neurons from rhesus monkeys showed a relationship between increased GnRH gene expression and decreased CpG methylation status, confirming the inhibitory role of DNA methylation in the timing of puberty (45). Evidence of changes in methylation has also been confirmed in humans, where distinct methylation states have been found to be associated with sex (46) and adolescent transition (47). Up to now, there are no data reporting defects in MKRN3 methylation in patients with diagnosis of CPP (48). Bessa et al. conducted the only methylome study in patients with CPP in 2018 (49) on peripheral blood leukocytes. The data obtained showed different methylation profiles in normal and precocious puberty with more than 80% differentially methylated CpG sites (DMS). These methylation changes affected several ZNFs genes, supporting the hypothesis of involvement of transcriptional repressors containing ZNF (zinc finger) motifs in human pubertal development, as previously demonstrated in nonhuman primates (49). Although a hypermethylation of *MKRN3* in pubertal or CPP subjects, no differences have been found between controls and CPP patients. Instead, the hypomethylated ZFP57 protein has been shown to be required for normal imprinting of genomic regions that control MKRN3 expression (49). No conclusions can be drawn from this single piece of data, which should instead urge the scientific community to further investigate the methylation status of MKRN3 in humans.

In addition to DNA methylation, other epigenetic regulatory mechanisms such as those mediated by microRNAs (miRNAs) are known to play key roles in the regulation of a wide range of cellular and body functions. A recent integrated analysis of DNA methylation and miRNA expression identified the influence of these genetic regulators on complex traits, such as age at menarche (50). Although the potential involvement of miRNAs in the control of MKRN3 activity has not been thoroughly studied, it has recently been reported that the 3’ UTR of the Mkrn3 transcript is a key element in the post-transcriptional regulation of miRNA-mediated gene expression. This recent evidence suggests a novel and interesting mechanism of puberty regulation upstream of MKRN3.

### MKRN3 in PWS and defects in methylation state

The human *MKRN3* gene is associated to Prader Willi syndrome, which results from genomic imprinting errors with lack of expression of paternally imprinted genes located in the 15q11.2-q13 region, as previously mentioned. The genetic mechanism commonly responsible for this disorder is the deletion of a 5-6 Mb region of paternally imprinted chromosome 15 (found in 65-75% of affected individuals). The remaining individuals have a maternal uniparental disomy (UPD15), or sporadic defects in a genomic region that controls the imprinting process. Despite this syndrome has been extensively investigated, the functional and physiological relevance of *MKRN3* in PWS is still not fully elucidated although this gene is now clearly associated with developmental processes. Indeed, it is interesting to note that the hypogonadism typical of PWS manifests with incomplete or delayed pubertal development and that only in a small number of subjects is the lack of the paternal *MKRN3* allele associated with CPP (51, 52).

A recent pilot study conducted by Mariani et al. (53) reported MKRN3 detectable serum levels in half patients in a cohort of genetically confirmed PWS, regardless of the genetic etiology of the syndrome (del or UPD).

Even MKRN3 serum levels in PW subjects were lower than those typically reported in the literature for normal as well as CPP patients, this finding suggests residual expression of the theoretically silenced maternal allele or its reactivation by demethylation. Prior to this study, it was widely accepted that only the paternal allele of *MKRN3* was expressed, while the maternal allele was completely silenced. However, in the brains of mice with deletion of the imprinting center, incomplete silencing of the genes included in the paternally inherited PWS critical region and a low level of expression of the maternal alleles were reported (54). In addition, gene expression studies by Rogan et al. reported loss of imprinting in the lymphoblasts of two PWS patients with a deletion and two atypical PWS patients with a maternal dysomy. Although these studies were not extended to brain expression profiles, the transcription of a subset of imprinted genes in some patients with PWS with maternal UPD suggested that relaxation of imprinting in these patients could be responsible for milder phenotypes (55).

Although further studies are needed, similar mechanisms could be hypothesized for all genes within the PWS region, including *MKRN3*. Potentially, analyses of human genome sequence and global methylation in patients with PWS, with or without CPP, could establish epigenetic alterations of MKRN3 in the pathogenesis of this disorder.

### MBD3

The expression of DNA methyltransferases (DNMT) and methyl binding proteins (MBP) mRNA in goats and rats showed different expression between the prepubertal and pubertal stages, highlighting the involvement of these proteins in the epigenetic regulation of puberty (56).

In particular, proteins from the methyl-CpG binding domain (MBD) family as MBD2 and MBD3 interact with members of the nucleosome remodeling deacetylase complex to suppress gene expression. MBD3 has been shown to the bind primarily to gene promoter and support TET2 activity, suggesting that it could dynamically and specifically influence the methylation status of gene loci (57).

Protein interaction assays identified the MBD3 methyl-CpG-binding protein as a physiological substrate for the E3 ligase activity of MKRN3, which was shown to conjugate poly-ubiquitin chains to lysines on multiple sites in MBD3 (18). One of these sites resides in the methyl-CpG binding domain of MBD3 whose MKRN3-mediated ubiquitination could disrupt the binding of MBD3 to the GNRH1 promoter containing 5hmC. The other major sites for such ubiquitination were mainly located in the C-terminal fragment of MBD3, which is directly involved in the MBD3-TET2 interaction, suggesting that this polyubiquitination of MBD3 would impair the MBD3-TET2 interaction resulting in a decrease in 5hmC content in mammalian genomes, but could also affect the binding of MBD3 to its target loci, such as the GNRH1 promoter. These two mechanisms would, in concert, ensure the suppressive effect of MKRN3 on GNRH1 expression, thus inhibiting the initiation of puberty. This repression imposed by MKRN3 on GNRH1 expression would be lost when the action of MKRN3 is impaired by loss-of-function mutations, as demonstrated by the *in vivo* ubiquitination assay performed on immortalized B lymphocytes derived from patients with CPP with MKRN3 defect (18). Furthermore, wild-type MKRN3 has been shown to fail to suppress GNRH1 transcription in cells with genetically ablated endogenous MBD3, suggesting the existence and synergy of the MKRN3-MBD3 complex in the regulation of hypothalamic initiation of puberty. Reduced MKRN3 activity and impaired MBD3-TET2 interaction could promote demethylation in the GNRH1 promoter and hypothalamic activation of GNRH1 transcription with the consequent onset of puberty (**Figure 2**).

These results demonstrate a new molecular mechanism through which MKRN3 contributes to regulate the epigenetic switch in the onset of mammalian puberty.

### Mirna: Mir30b

There is still a conspicuous lack of knowledge about the mechanisms of upstream MKRN3 regulation. Using miRNA-target prediction tools based on different bioinformatics methods, several sets of potential miRNA regulators for the MKRN3 gene were identified. In particular, three regions in the 3’ UTR of MKRN3 were predicted to have a high affinity for members of the miR-30 family (i.e., miR-30a, miR-30b, miR-30c, miR-30d, and miR-30e). In the rat hypothalamus, the expression of Mkrn3 and miR-30b displayed opposite profile during postnatal maturation and co-expression in Kiss neurons and Kiss1 cell lines (mHypoA-55). The possible repressive role of miR-30b was demonstrated by functional *in vitro* analyses. Luc-Pair miR luciferase assays cotransfecting pre-miR-30b with the reporter plasmid containing the 3’UTR of Mkrn3 showed a marked reduction in the luciferase signal (>65%) indicate that miR-30b targets the 3’ UTR of Mkrn3 and drives a repressive signal to Mkrn3 expression *in vitro*. Furthermore, during the juvenile period, central infusion of miR-30 blockers that bind to the 3’ UTR of Mkrn3 reversed the prepubertal decrease in hypothalamic Mkrn3 protein and delayed female puberty.

## Conclusions

The extremely early activation of HPG is referred to as central precocious puberty (CPP). Studies on this disorder are useful in unraveling the mechanisms that regulate the onset of puberty. The increasing number of reported loss-of-function mutations in the MKRN3 gene has made it the most frequent monogenic cause of CPP. Although the function of this gene is not completely known, MKRN3 appears to function as a brake on neuronal GnRH release, preventing activation of the HPG axis. A number of studies have begun to point to possible targets of MKRN3 action; indeed, mass spectrometry analyses performed on a stable HEK cell line revealed 81 new protein interaction partners of MKRN3 with high reliability. Among these, 20 interactions were previously associated with age at menarche in GWAS studies. Functional studies, mainly based on luciferase assays and protein interaction analyses, have started to report results on possible mechanisms of action by which MKRN3 might mediate its effects. The first mechanism of action is related to its ubiquitin ligase activity. MKRN3 is in fact able to add ubiquitin chains both on its targets, by promoting their proteasome-mediated degradation, and on itself, by regulating its own expression levels. Essential for this function is the integrity of the RING FINGER domain in which the enzyme activity resides. MKRN3 has been shown to interact with the neural precursor prentraxin-1 (Nptx1) in the hypothalamus of 4-week-old mice, and the C3HC4 ring finger domain appears to be essential for this interaction. Furthermore, reduced Nptx1 polyubiquitination was found in the hypothalamus of mice injected intracerebroventricularly with the Mkrn3 mutant lacking the RING domain, which confirmed its ubiquitin-mediated inhibition. Similarly, MKRN3 modulates other effectors, such as PABPC1, involved in GNRH mRNA stabilization mechanisms, and MBD3, involved in GNRH promoter methylation. Again, mutations in the RING of MKRN3 report reduced inhibition of its targets, resulting in increased GNRH levels and the onset of puberty.

The second hypothesized mechanism of action sees MKRN3 as a transcriptional repressor. Studies on neuronal cell lines have indeed confirmed a direct interaction of MKRN3 with the promoters of genes involved in pubertal timing, such as *KISS1* and *TAC3*. The effects of some CPP-associated MKRN3 missense mutations revealed that only mutations located in the RING finger domain impair MKRN3’s ability to inhibit the activity of the KISS and TAC3 promoters, but not its recruitment. On the other hand, mutations located in the N- and C-terminal domains of MKRN3 showed reduced binding affinity to the promoters of the aforementioned genes, without compromising its inhibitory activity. This supports the hypothesis that MKRN3 does not act directly as a transcriptional regulator of these genes, but rather indirectly, possibly as part of the transcriptional repressor network, as already demonstrated for Mkrn1.

Finally, epigenetic mechanisms have also been implicated in regulating the onset of puberty. In particular, very recently miRNAs have emerged as novel CPP-associated factors that contribute to the regulation of both kisspeptin and GnRH secretion. The latest evidence shows that miR-30b drives a repressive signal for Mkrn3 expression *in vitro* by binding to its 3’ UTR.

As can be seen from the studies reported in this review, there are multiple ways through which MKRN3 exerts its action on puberty onset timing.

Studies on defects in this gene causing CPP in humans have helped to elucidate these mechanisms.

Further studies are needed to investigate new effectors of MKRN3 and to confirm the mechanisms of action postulated so far.

## Author contributions

SP wrote the manuscript and designed the figures. GC wrote the manuscript. FA, AP, and EM made a critical revision of the manuscript. AG supervised the manuscript and final approval. All authors contributed to the article and approved the submitted version.

## Funding

This work was supported by a grant (390) funded by “VALERE: VAnviteLli pEr la RicErca” program of University of Campania “L. Vanvitelli”.

## Conflict of interest

The authors declare that the research was conducted in the absence of any commercial or financial relationships that could be construed as a potential conflict of interest.

## Publisher’s note

All claims expressed in this article are solely those of the authors and do not necessarily represent those of their affiliated organizations, or those of the publisher, the editors and the reviewers. Any product that may be evaluated in this article, or claim that may be made by its manufacturer, is not guaranteed or endorsed by the publisher.
